# Autotaxin–Lysophosphatidate Axis: Promoter of Cancer Development and Possible Therapeutic Implications

**DOI:** 10.3390/ijms25147737

**Published:** 2024-07-15

**Authors:** Carmelo Laface, Angela Dalia Ricci, Simona Vallarelli, Carmela Ostuni, Alessandro Rizzo, Francesca Ambrogio, Matteo Centonze, Annalisa Schirizzi, Giampiero De Leonardis, Rosalba D’Alessandro, Claudio Lotesoriere, Gianluigi Giannelli

**Affiliations:** 1 Medical Oncology Unit, National Institute of Gastroenterology, IRCCS “S. de Bellis” Research Hospital, 70013 Castellana Grotte, Italy; 2 Medical Oncology, IRCCS Istituto Tumori “Giovanni Paolo II”, Viale Orazio Flacco 65, 70124 Bari, Italy; 3 Section of Dermatology and Venereology, Department of Precision and Regenerative Medicine and Ionian Area (DiMePRe-J), University of Bari “Aldo Moro”, 70124 Bari, Italy; 4 Personalized Medicine Laboratory, National Institute of Gastroenterology, IRCCS “S. de Bellis” Research Hospital, 70013 Castellana Grotte, Italy; matteo.centonze@irccsdebellis.it; 5 Laboratory of Experimental Oncology, National Institute of Gastroenterology, “IRCCS “S. de Bellis” Research Hospital, 70013 Castellana Grotte, Italy; annalisa.schirizzi@irccsdebellis.it (A.S.); giampiero.deleonardis@irccsdebellis.it (G.D.L.);; 6 Scientific Direction, National Institute of Gastroenterology, IRCCS “S. de Bellis” Research Hospital, 70013 Castellana Grotte, Italy

**Keywords:** autotaxin (ATX), *ENPP2*, lysophospholipase D (lysoD), lysophosphatidic acid (LPA), lysophosphatidylcholine (LPC), ATX-LPA axis, tumor microenvironment, cancer

## Abstract

Autotaxin (ATX) is a member of the ectonucleotide pyrophosphate/phosphodiesterase (*ENPP*) family; it is encoded by the *ENPP2* gene. ATX is a secreted glycoprotein and catalyzes the hydrolysis of lysophosphatidylcholine to lysophosphatidic acid (LPA). LPA is responsible for the transduction of various signal pathways through the interaction with at least six G protein-coupled receptors, LPA Receptors 1 to 6 (LPAR1–6). The ATX–LPA axis is involved in various physiological and pathological processes, such as angiogenesis, embryonic development, inflammation, fibrosis, and obesity. However, significant research also reported its connection to carcinogenesis, immune escape, metastasis, tumor microenvironment, cancer stem cells, and therapeutic resistance. Moreover, several studies suggested ATX and LPA as relevant biomarkers and/or therapeutic targets. In this review of the literature, we aimed to deepen knowledge about the role of the ATX–LPA axis as a promoter of cancer development, progression and invasion, and therapeutic resistance. Finally, we explored its potential application as a prognostic/predictive biomarker and therapeutic target for tumor treatment.

## 1. Introduction

Autotaxin (ATX) is a glycoprotein, a member of the ectonucleotide pyrophosphate/phosphodiesterase (*ENPP*) family [1]. *ENPP2* is the gene that encodes for ATX; its expression depends on growth factors, cytokines, and hormones [2]. Secreted ATX acts as a lysophospholipase D (LPD), converting extracellular lysophosphatidylcholine (LPC) into lysophosphatidic acid (LPA). LPA has growth factor-like activity and is responsible for the transduction of various signal pathways through its interaction with at least six different G protein-coupled receptors, LPA Receptors 1 to 6 (LPAR1–6) [3]. The degradation of LPA in monoacylglycerol (MAG) is regulated by lipid phosphate phosphohydrolase (LPP1–3) localized on plasma membranes. The role of LLP1–3 is fundamental in moderating the LPA biological effects [4,5].

ATX and LPA can be detected in human blood, although they mainly perform a local action rather than a systemic activity in consideration of their short half-life in circulation [2].

The ATX–LPA axis is responsible for several physiological and pathological effects [5,6,7,8,9]. For example, this signal pathway is involved in embryogenesis, angiogenesis, lymphangiogenesis, and wound healing [10]. On the other hand, several pathologies are linked to an aberrant ATX–LPA axis, such as inflammation, fibrosis, and obesity. Furthermore, relevant research demonstrated its pivotal role in tumor growth [6,7,8]. In detail, aberrant ATX–LPA signaling is correlated to carcinogenesis, immune escape, metastasis, tumor microenvironment (TME), cancer stem cells, and therapeutic resistance [11].

In this review of the literature, we aimed to deepen knowledge about the role of the ATX–LPA axis as a promoter of cancer development, progression and invasion, and therapeutic resistance. Finally, we explored its potential application as a prognostic/predictive biomarker and therapeutic target for tumor treatment.

## 2. Autotaxin

### 2.1. Structure and Isoforms

The human ATX gene contains 26 introns and 27 exons and is located at position 8q21.q on chromosome 8 [2]. It is considered a potential susceptibility locus for tumor genesis. Based on different forms of mRNA alternative splicing, five isoforms of ATX are known and can be found in various tissues. The isoforms are called α, β, γ, δ, and ε and differ according to the presence or not of sequences codified by exons 12 and 21. These variants have differences in stability and functions although they are not well defined yet. However, all of them have lysophospholipase D (lysoPLD) activity.

ATXα is characterized by the deletion of exon 21 and is expressed in the peripheral tissues and central nervous system (CNS). ATXβ is an isoform in which exons 12 and 21 are deleted; it is present in human peripheral tissues at significantly higher levels than ATXα. ATXγ presents exon 12 deletion and is widely expressed in the brain and CNS. ATXδ and ATXε are isoforms in which four amino acids are deleted in the L2 linker region of ATXβ and ATXα, respectively. Among all these variants, ATXβ and ATXδ are the most stable and represented in several organisms [12,13,14].

From a structural point of view, ATX is a type II ectonucleotide of 125 kDa, composed of seven ectoenzymes [15]. ATX structure consists of a central phosphodiesterase (PDE) domain that interacts, on one side, with the catalytically inactive nuclease (NUC)-like domain in the C-terminus, and on the other side with two somatomedin B (SMB1-2)-like domains in the N-terminus [16,17]. PDE and NUC domains are linked by a disulfide bond; the NUC domain is surrounded by a lasso, which keeps the PDE domain rigidity. The PDE domain consists of two zinc ions, a catalytic site, and a deep hydrophobic lipid pocket responsible for the binding with different types of LPC and LPA [16,17]. The SMB1-2 domain has a peculiar structure, enriched with cysteine, which interacts with adhesive molecules like integrins at the cell surface. In this way, the ATX activity is focused on the cell surface, next to LPA receptors (LPARs), so that LPA can be directly released to the target receptors [18,19]. Therefore, ATX, full of LPA, can bind to the exosome surface and be delivered to the target cells, where it interacts with cell surface integrins. There, ATX can control specific signaling in the target cells through the regulation of LPA production or degradation [18,19].

### 2.2. Regulation of Autotaxin Expression

*ENPP2* is expressed in several tissues, such as adipose tissue and the brain, but it is also found in high concentrations in different biological fluids, in particular in serum and blood [20]. The production of LPA, the activation of its receptors, and the consequent downstream signaling are strictly dependent on ATX expression. Nowadays, the mechanism of regulation of ATX production is not well-known, because several mechanisms have been reported in the literature [2,15].

Previous research findings suggest that ATX undergoes intricate regulation at both the transcriptional and post-transcriptional stages. Notably, treatment with histone deacetylase (HDAC) inhibitor proved to induce ATX expression in different tumor cells [21]. The ATX promoter was characterized by H3K27me3 marks in HEK 293T cells, lately. Interestingly, expression levels of ATX were found to increase following lipopolysaccharide (LPS) therapy, facilitated by the demethylase UTX-mediated removal of methyl groups from H3K27me3. This observation implies that epigenetic alteration plays an important role in regulating ATX expression. Additionally, a distant enhancer has been found as a key regulator of ATX expression. Through enhancer-mediated interactions with the ATX locus, JMJD3-DDX21 prevents R-loop formation and promotes transcript synthesis following exposure to LPS [22].

Numerous transcription factors play a critical role in influencing ATX transcriptional regulation. For instance, the reduction of the nuclear transcription factor of activated T cell 1 (NFAT1) expression also leads to the decreased expression of ATX, resulting in the suppression of tumor growth and metastasis in melanoma models [23]. ATX promoter can be also regulated following the activation of a GA-box and a cAMP-Response Element-binding protein/Activator Protein-1 (CRE/AP-1)-like element by the transcription factors Specifity Protein (SP) and AP-1 [24]. Moreover, a recent study showed that the migration of breast cancer (BC) cells can be enhanced as a result of ATX activation mediated by the transcription factor Stat3 (Signal transducer and activator of transcription 3) [25]. In vitro studies by Michelle et al. demonstrated that hypoxia enhances ATX expression in hepatocellular carcinoma (HCC) via Hypoxia-inducible factor-1 (HIF-1) as well as hepatitis C virus infection, favoring liver injury and fibrosis [26]. Other experimental data reported that the transcription factors c-jun, v-jun, and Homeobox A13 (HOXA13) are involved in ATX expression [27,28,29].

Recent research has unveiled post-transcriptional mechanisms affecting ATX regulation. AU-rich element RNA binding protein 1 (AUF1) and Human antigen R (HuR) are RNA-binding proteins that specifically interact with the ATX mRNA 3’-UTR, influencing ATX mRNA stability in LPS-stimulated THP-1 human monocytic cells and Colo320 human colon cancer (CR) cells [30]. The well-known oncosuppressor microRNA-101-3p (miR-101-3p) inhibits ATX expression by binding a specific sequence in the ATX mRNA 3’-UTR [31]. In addition, a recent experiment by Zhang et al. revealed that RNA methyltransferase Nsun2 methylates the 3’-UTR of ATX mRNA at cytosine 2756. Consequently, Nsun2-mediated methylation enhances the export of ATX mRNA towards the cytoplasm, favoring ATX translation [32].

Various inflammatory cytokines can also impact ATX regulation. For instance, TNF positively influences ATX transcriptional expression [33]. Toll-like receptor (TLR) ligands, such as LPS, poly(I:C), and CpG oligonucleotide, can induce ATX expression in THP-1 cells [34].

The initial form of ATX is a pre-pro-enzyme with a 27-residue signal peptide in the N-terminus. ATX secretion depends on the hydrophobic core sequence within the signal peptide and the N-glycosylation at amino acids N410 and N53 [35]. ATX passes from the endoplasmic reticulum (ER) to the cytoplasm through a process that involves p23 and Sec24C under the control of the Protein kinase B - Glycogen synthase kinase-3 (AKT-GSK-3) signaling pathway [36]. Lyu et al. demonstrated that a di-phenylalanine motif (Phe-838/Phe-839) located in a region of ATX at the C-terminus plays a pivotal role in the interaction between this protein and p23 [36].

On the other hand, the reduction of ATX expression correlates with elevated LPA level in the circulation [37,38]. Furthermore, other research has revealed that liver sinusoidal endothelial cells (LSECs) play a key role in clearing ATX from circulation and facilitating its degradation via the scavenger receptor [39].

Finally, other studies have examined how the epigenetic alterations of *ENPP2* gene could influence its expression. In particular, DNA methylation is the most studied epigenetic change. It corresponds to the addition of a methyl group selectively within the 5 position of the cytosine ring. This phenomenon regards CpG dinucleotides (CGs) that are known as “CpG islands” [40,41]. A significant methylation of the *ENPP2* gene was observed in several healthy human tissues [20]. In detail, a high level of methylation was detected in the gene body CGs and a low level of methylation in the promoter and the 1st exon CGs [40,41]. On these bases, a low level of promoter methylation could be correlated to the active transcription of *ENPP2* in healthy tissues.

### 2.3. Functions

ATX acts as a lysoPLD, it is the only member with this type of activity in the context of the *ENPP* family [2]. ATX is primarily responsible for the hydrolyzation of extracellular LPC, resulting in the production of LPA and choline. Moreover, ATX can also hydrolyze phosphosphingolipids, such as sphingosylphosphorylcholine (SPC) into sphingosine 1-phosphate (S1P). However, ATX has a higher affinity for LPC, resulting in distinct signaling outcomes based on the generated products [38,42,43].

## 3. LPA and LPAR1–6

LPA species are a family of small glycerophospholipids characterized by a phosphate head group and a glycerol backbone linked to a fatty acid chain at the sn-1 or -2 position. The various LPAs differ by the length and saturation of the acyl or alkyl fatty acid chain [44,45].

LPA production depends on ATX activity that can begin from lysophosphatidylcholine linked to albumin in serum or from membrane lysophospholipids [46]. LPA is rapidly cleared from the circulation (t_1/2_ of around 1 min) by three lipid phosphate phosphatases (LPP1–3) [47]. The plasma concentration of LPA directly influences the downregulation of ATX mRNA, while it has a weak inhibitory impact on ATX activity [37].

LPA plays a pivotal role in several physiologic and pathologic conditions. For instance, it is involved in the correct development of neural crest, tissue remodeling, and wound healing through the promotion of numerous cellular functions. In fact, LPA can stimulate cell proliferation, survival, and migration [3,48,49,50].

LPA performs its functions through the binding to specific G protein-coupled receptors (GPCRs) located at the cell membrane. Six LPARs have been identified, called LPAR1–6. They transmit the signals by means of activation of the G_i_, G_s_, G_q_, or G_12/13_ subunits. This event induces different downstream signaling pathways, such as RAS, PI3K, Rho, and PLC [3]. The *LPAR1, 2*, and *3* genes belong to the group of endothelial differentiation gene (*EdG*); *LPAR4* and *5* are classified in the purinergic group. *LPAR6* was recently discovered, and it is also known as GPC receptor P2Y5. The expression of LPAR1–6 is variously distributed in the body tissues [51]. In detail, it can be found in the lungs, spleen, brain, small intestine, stomach, heart, kidney, uterus, and placenta. Otherwise, some LPARs are restrictively expressed in some organs: LPAR2 in the kidneys, testis, uterus, and leukocytes; LPAR3 in the pancreas, prostate, and heart; LPAR4 in the ovary [48,52].

**Figure 1:**
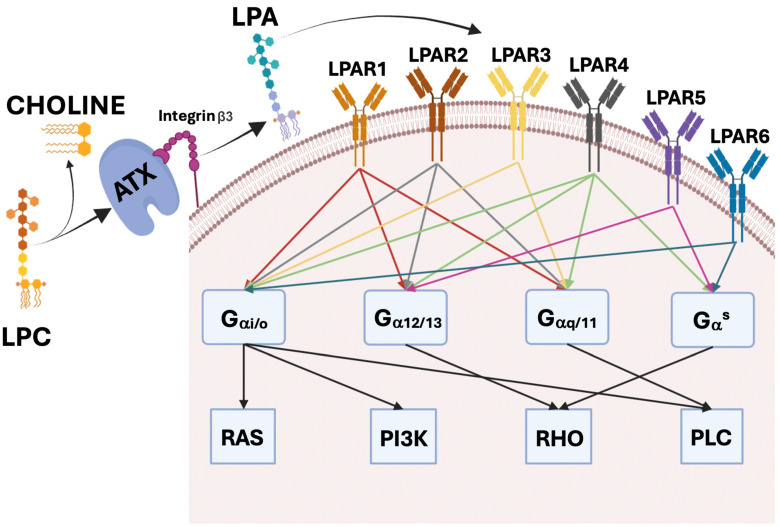
Representation of the ATX–LPA axis. ATX is linked to plasmatic membrane by means of Integrinβ3, near to LPA receptors. ATX converts LPC in LPA, which, in turn, binds to LPAR1–6 very efficiently in consideration of the proximity. Consequently, the LPA-mediated activation of LPAR receptors induces different downstream signaling pathways, including RAS, PI3K, Rho, and PLC through different G proteins. Each LPAR (1–6) activates specific G proteins and it is indicated by the same color lines of the receptor.

LPA synthesis can also occur intracellularly; in this regard, it exerts its activity through an intracellular LPAR, the nuclear receptor peroxisome proliferator-activated receptor gamma (PPARγ) [53] (Figure 1).

## 4. ATX–LPA Axis in Physiological Conditions

High levels of ATX expression were found during the different phases of embryogenesis and in adults. In mouse models, ATX expression is mainly present in the anterior portion of the neural tube and the midbrain at E8.5, in the floor plate of the neural tube at E10.5, in intestines and glands at E11.5–12.5, in muscle tissues and mesenchymal kidney at E13.5 and E16.5, in epithelial cells of choroid plexus from E13.5 to birth [54].

Numerous experimental data underlined the importance of ATX for embryogenesis. Embryonic lethality was observed in homozygous ATX-knockout mice due to the formation of weak blood vessels for structural defects in the neural tube and extraembryonic yolk sac. On the other hand, good embryonic development was reported in heterozygous ATX-knockout mice [55].

With regards to adults, abundant ATX was found in various organs including the central nervous system (CNS), adipose tissue, placenta, and lymph nodes but also in several biological fluids, such as cerebrospinal liquid, blood, seminal fluids, and urine [56,57].

The high levels of ATX in adipose tissue suggest a strong correlation between this glycoprotein and lipid metabolism. Recent evidence indicated that a high amount of ATX is present during the process of differentiation in preadipocytes cell lines 3T3-F442A, 3T3-L1 and primary preadipocytes [58,59,60]. On the other hand, the mouse model with the disruption of ATX in adipose tissue showed a reduction of about 38% of circulant LPA levels. Other studies showed a possible correlation between ATX and the impairment of glucose homeostasis. Zhang et al. demonstrated that a group of cytokines including IL-6, ciliary neurotrophic factor (CNF), leukemia inhibitory factor (LIF), and cardiotrophin-1 increased gp130-mediated ATX expression in adipose tissue. The blockade of gp130 signaling determined the suppression of ATX expression improving insulin sensitivity [58].

Experimental studies have found that inflammation promotes the expression of ATX, which in turn stimulates inflammation in obese subjects through the induction of specific pro-inflammatory cytokines. Thus, in adipose-specific ATX-deficient mouse models, a significant reduction of IL-6, MCP-1, and TNF in circulation and adipocytes was reported [61].

A possible correlation has also been described of the ATX–LPA axis with the occurrence of atherosclerosis [62,63,64], since LPA also derives from the oxidative modification of low-density lipoproteins (LDLs), which are implicated in the forming of atherosclerotic plaques. Moreover, LPA can stimulate the secretion of various chemokines (cxcl-1, MCP-1), cytokines (IL-1, IL-8), and adhesion molecules (VCAM-1, E-selectin) by endothelial cells (ECs). In this way, LPA favors the adhesion of lymphocytes to ECs [65,66,67]. Furthermore, the ATX–LPA axis can increase endothelial permeability and lead to plaque instability, promoting the deterioration of atherosclerosis [68,69,70].

ATX is also involved in the regulation of the immune system. In fact, this glycoprotein can bind to lymphocytes, promoting the migration of T cells from blood, through the endothelial barrier, into lymphoid tissues [57,71].

In addition, it seems that ATX can be also produced by placental trophoblasts during pregnancy [72,73,74]. In this regard, it has also been reported that serum levels of ATX increase in direct proportion with gestational weeks [72,73,74]. A reduction of placental ATX mRNA levels was reported in impaired pregnancies. At the same time, the serum levels of ATX rapidly decrease to the nonpregnant state after delivery [72,73,74].

## 5. ATX–LPA Axis in Cancer

### 5.1. The Role in Cancer Genesis, Invasion, and Metastasis

Experimental data reported that the ATX–LPA axis could directly induce oncogenic alterations in healthy cells. In detail, Liu et al. demonstrated that the forced expression of ATX or one among LPAR1, LPAR2, and LPAR3 in the mammary glands of transgenic mouse models led to the occurrence of BC [75]. The modulation of LPAR expression is also correlated with cancer genesis. In pancreatic duct adenocarcinoma (PDAC), the mRNA levels of LPAR1 are lower than in normal pancreatic tissue (NPT) in contrast with mRNA levels of LPAR3 that were higher in PDAC. This evidence supports the hypothesis that the down- and upregulation of LPAR1 and LPAR3, respectively, are correlated with the tumor transformation of the pancreatic duct cells [76].

Other studies on mouse models showed that tumor aggressiveness can be enhanced by the autocrine activity of the ATX–LPA axis. In particular, the injection of ATX-transfected NIH-3T3 fibroblast cell lines into nude mice showed a more invasive and metastatic behavior of these cells than control ones [77]. In addition, the disruption of *ENPP2* in mouse PDAC significantly decreased the proliferative potentiality of PDAC cells. Moreover, mice that were subjected to the injection of *ENPP2* ko PDAC cells showed impaired cancer growth [77]. In this regard, we discovered that *ENPP2* expression was upregulated in PDAC and in HCC with respect to normal tissue. The same result was found for cholangiocarcinoma (CCA), although the difference was not significant compared to normal liver tissue [78]. In this experiment, we also demonstrated that the ATX inhibition through IOA-289 treatment on different tumor cell lines of Prostatic Cancer (PC) (Panc-1, MIA PaCa-2), HCC (HLF, HLE), CCA (RBE, KKU-M213), and Colorectal Cancer (CRC) (HT-29, Caco2) led to reduction of cell proliferation and migration [78]. Moreover, IOA-289 treatment was also responsible for the stimulation of the apoptosis through the up regulation of numerous proteins, including Caspase-3 and -8, BIM, and FAS [78].

On the other hand, the ATX–LPA axis is also involved in tumor progression. In fact, several in vitro and in vivo data reported that the LPA signaling pathway plays a relevant role in tumor proliferation, growth, and invasiveness. However, the effects of LPA depend on the types of tumor cells and expressed LPAR. Indeed, the activation of LPAR1 and LPAR3 induces opposite effects on pancreatic tumor cells [76]. To be specific, LPAR3-deficient hamster PDAC cells have a lower capacity in terms of motility, invasiveness, and proliferation than LPAR1-deficient PDAC cells. In another experimental study, the proliferative activity of human ovarian tumor cells was correlated to the activation of LPAR2 and LPAR3 signaling pathways but not LPAR1. On the other hand, invasion and motility were dependent on LPAR1, LPAR2, and LPAR3 signaling pathways [79].

High levels of ATX, LPA, and LPAR1 expression were detected in HCC. Recent data on this primary liver cancer demonstrated that LPA leads to an increased expression of Matrix Metalloproteinase-9 (MMP-9) and an enhanced tumor cell invasiveness by means of LPAR1 and the combined activation of the p38-MAPK (Mitogen Activated protein Kinase) and phosphoinositide 3-kinase (PI3K) signaling pathways [80,81]. Sabbà et al. demonstrated that overexpression of LPAR6 favors HCC genesis and growth. Moreover, aberrant expression of this receptor was correlated with poor prognosis in a cohort of 128 HCC patients [82].

The ATX–LPA axis also mediates the prometastatic activity, although different effects can be obtained according to the type of tumor cell, the cancer stage, and the LPAR involved. In a mouse model of BC, the forced expression of LPAR1, LPAR2, or LPAR3 led to enhanced invasive and metastatic activities. The mice that overexpressed LPAR3 have a higher rate of BC metastases than those mice overexpressing LPAR1 or LPAR2 [75]. In this regard, Popnikolov et al. conducted a study on 87 human BCs in which they demonstrated that the expression of ATX and LPAR3 was significantly higher in mammary tumor tissues than in normal breast ones. Moreover, these authors showed that advanced-stage and lymph node metastases were more often present in LPA3-expressing BCs than in LPAR3^−^ cancers [83]. Other mouse models of BC evidenced that the LPAR1 signaling pathway was responsible for metastatic activity. The administration of Debio-0719, a highly specific LPAR1 antagonist, reduced tumor cell migration to the lungs and bones [84].

**Figure 2:**
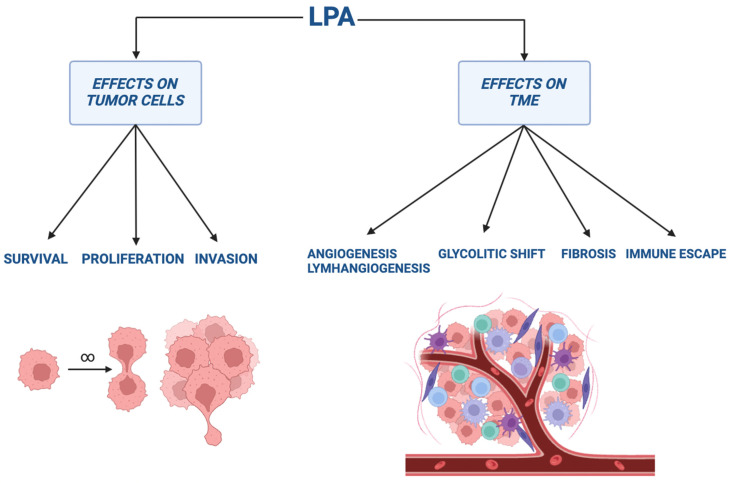
Schematic description about LPA effects on tumor cells and tumor microenvironment.

From a clinical point of view, the circulant level of LPA could be considered a biomarker of cancer progression. In this regard, it was discovered that the serum level of LPA was higher in HCC patients than in those with normal liver function or liver cirrhosis. In addition, serum levels of LPA in HCC patients were higher in cases with metastasis, larger HCC tumors, and shorter survival [85] (Figure 2).

### 5.2. Stimulation of Angiogenesis and Lymphangiogenesis

Angiogenesis and lymphangiogenesis play a pivotal role in tumor growth, genesis of the TME, and metastatization [86]. Several experimental studies showed that ATX may exert proangiogenic and pro-lymphangiogenic actions, although not for all types of tumors.

Nam et al. reported that the transfection of ATX-transfected/Ras-transformed NIH-3T3 cells mixed with Matrigel into nude mice led to the genesis of new blood vessels in larger amounts than the controls [77]. On the other hand, ATX alone mixed with Matrigel showed a comparable proangiogenic activity to vascular endothelial growth factor (VEGF) [87]. Experimental data evidenced that the stimulation of LPAR2–3 by LPA results in VEGF production in ovarian tumor cells [88]. Moreover, a strong relationship was found between VEGF level and LPAR2–3 in human ovarian cancer biopsies [89]. In addition, LPA can stimulate cancer-associated fibroblast (CAF)-mediated angiogenesis. In detail, CAFs may secrete VEGF and stromal cell derived factor-1 (SDF-1) which are well-known pro-angiogenic factors [90]. However, the same results were not observed in other types of tumors, such as BC [91]. In detail, the cell model of BC documented that LPA stimulation did not conduct VEGF production.

In the context of the VEGF protein family, VEGF-C is the member with the most important lymphangiogenic factor. It can stimulate lymphangiogenesis through the bond to VEGFR-3 [92]. A study on prostatic cancer (PC)-bearing mice reported that the employment of a specific VEGFR-3 antagonist determined the inhibition of lymphangiogenesis and dissemination of metastases [93]. VEGF-C expression can be also stimulated by LPA through the bond to LPAR1 and LPAR3 [94]. ATX and VEGF-C were found in large amounts in PC biopsies and were correlated with a high number of lymphatic vessels and a higher Gleason score [95]. To note, the pharmacological inhibition of LPAR1/LPAR3 with Ki16425 in PC-bearing nude mice led to a considerable reduction of lymphatic vessels and lymph node metastases in comparison with controls [95] (Figure 2).

### 5.3. The Effect on Tumor Glycolytic Shift and Fibrosis Development

Hypoxia is one of the main features of TME [96]. It is due to the persistent outgrowth of tumor cells leading to the formation of new vessels but with a deficient structure and poor oxygen supply [97]. Hypoxia induces the production of the hypoxia-inducible factor 1 (HIF1) that favors angiogenesis and influences the tumor cell metabolism. In detail, HIF1 can increase glycolysis and enhance lactate metabolism by favoring intra tumoral acidosis through the accumulation of H+ [98].

Recent data on ovarian cancer cell lines showed that LPA can stimulate a metabolic conversion towards glycolysis [99]. In particular, the activation of LPAR by LPA leads to the stimulation of the RAC-NOX-ROS pathway with the consequent elevation of HIF1. The latter induces the glycolytic enzyme hexokinase 2 (HKII) and glucose transporter-1 (GLUT1) expressions [99]. Moreover, another study documented that LPA can induce a metabolic shift toward glycolysis also in CAFs isolated from ovarian cancers [100].

Cancer associated fibroblasts (CAFs) derive from the differentiation of stellate cells, tumor-related adipocytes, or resident fibroblasts following the stimulation of PDGF, TGFβ, or FGF-2 [100,101,102,103,104,105,106]. The TGFβ shapes the TME, regulates the activation of various leukocyte subtypes and the differentiation of CAFs [106,107,108,109,110,111]. The desmoplasia in tumor tissue is strictly related to CAF action [112]. In contrast with common fibroblasts, CAFs are continuously activated and very resistant to apoptosis. CAFs have the function to produce the structure components of TME, such as fibronectin and collagens [113]. They also favor cancer invasion and metastasis through the action of MMP-2 and MMP-9 [114,115]. CAFs can release osteopontin and SDF-1, which stimulate tumor cell proliferation, but also produce renomedullin and VEGF that induce angiogenesis [90,116].

A lot of data evidenced that LPA can act as a CAF-generating factor. In this regard, an in vitro study documented that the LPA stimulation of human common fibroblasts led to their conversion into CAFs [100]. Moreover, Mazzocca et al. also demonstrated that HCC cells can secrete LPA favoring the trans differentiation of peritumoral tissue fibroblasts (PTFs) to a CAF phenotype [85]. Consequently, PTFs increased tumor cell proliferation, migration, and invasion. On the other hand, the silencing of the *ENPP2* gene or the treatment with α-bromomethylene phosphonate [BrP]-LPA, a pan-LPA inhibitor, inhibited this PTF trans differentiation and the consequent effects on HCC cells [85]. Furthermore, Jeon et al. reported that human adipose tissue-derived mesenchymal stem cells (hASCs) can differentiate into CAFs by means of the conditioned medium from ovarian tumor cell lines [90]. These differentiations need the LPA-mediated activation of several signaling pathways including various factors, such as phosphoinositide-3-kinase (PI3K), Rho-kinase, ERK, and PLC [90]. TME-associated CAFs are the most important producers of collagen in tumors. An elevated deposition of collagen in tumor stroma is responsible for desmoplasia [117]. Moreover, these collagens have often a crosslinked structure, leading to a disruption of tumor tissue architecture. This alteration determines an increased pressure of interstitial fluid and a reduction of drug delivery to tumors with a possible therapeutic resistance. In addition, poor clinical results were observed in those patients affected by tumors with a severe CAF-derived desmoplastic reaction [117] (Figure 2).

### 5.4. Immune Escape

It is well known that tumors can implement mechanisms to evade the immune system so that they can develop undisturbed. In particular, TME plays a pivotal role in tumoral immune escape thanks to its ability to prevent T lymphocytes from penetrating inside the tumor itself. In this way, the T lymphocytes cannot carry out their effector functions [118]. On the other hand, the TME hosts many tumor-associated macrophages (TAMs). These cells have anti-inflammatory functions and are usually correlated with a poor prognosis [119]. Recent data on ascites of ovarian cancer patients documented that TAMs express ATX and can produce large amounts of LPA. It has been also described that TAMs express LPAR3, LPAR5, and LPAR6, although with unknown functions [120]. According to some recent studies, LPA could also act as a TAM-converting factor, thanks to the ability to stimulate the conversion of circulating monocytes into macrophages [121].

The infiltration and activation of CD8+ cytotoxic T lymphocytes is a crucial phase in immune eradication [122]. In this regard, LPA can inhibit the activation of CD8+ T cells, helping cancer cells to avoid the immune system. Commonly, T- cell receptor (TCR) stimulation of CD8+ T cells lead to intracellular calcium mobilization and ERK activity [123,124]. However, the binding of LPA to LPAR5 on CD8+ T lymphocytes determine the inhibition of these processes. Moreover, LPA-mediated LPAR5 stimulation is responsible for the suppression of antigen-specific CD8+ T cell activation and proliferation, as well as for the inhibition of cytotoxic activity of these lymphocytes by blocking the exocytosis of granules with granzyme B [123,124]. To note, a study on mice with melanoma demonstrated that the treatment with LPAR5^−/−^ tumor-specific CD8+ T lymphocytes led to an elevated infiltration of CD8+ T cells in tumors. As a result, a significant reduction in cancer growth was observed [111,112].

However, other data suggest that the ATX–LPA axis could promote the T lymphocyte transmigration across the endothelium of blood vessels so that these cells could reach lymphoid organs and inflamed areas [125]. In this regard, Aiello et al. showed that ATX inhibition decreased allograft infiltration of CD4+ and CD8+ T cells in a rat model of fully allogeneic kidney transplantation [126]. In addition, Takeda et al. reported that the interaction between LPA and LPAR2 on naïve T lymphocytes is fundamental for the activation of the ROCK/myosin IIA pathway. In turn, this protein system improved T-cell migration across very small pores [127] (Figure 2).

## 6. The Role of ATX–LPA Pathway in the Different Cancers

### 6.1. Breast Cancer

**Figure 3:**
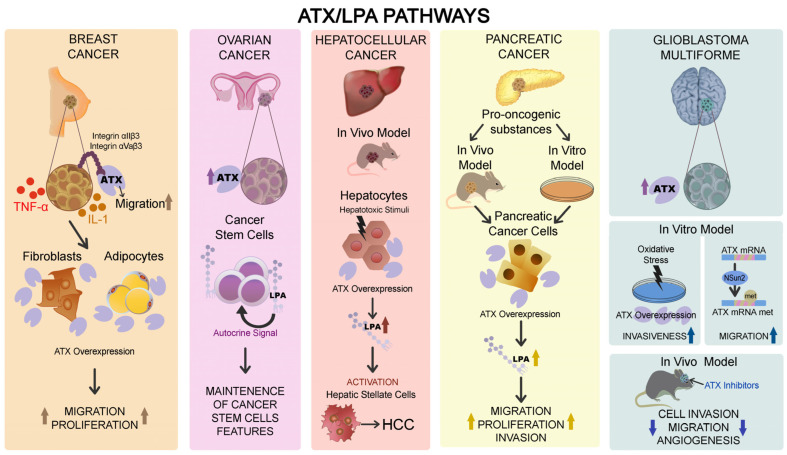
Schematic illustration of ATX–LPA pathway involvement in genesis and progression of breast cancer, ovarian cancer, hepatocellular cancer, pancreatic cancer, and glioblastoma multiforme.

BC is the most frequent tumor in women, with a 5-years survival rate of 80–85% [128]. It has been reported that ATX levels in circulation were significantly higher in BC patients than in healthy people [129]. BC cells commonly produce a low amount of ATX in contrast to melanoma and glioblastoma cells [130,131,132]. On the other hand, ATX is also secreted by BC-related fibroblasts and mammary adipose tissue [133]. In detail, BC cells can secrete several inflammatory mediators, including IL-1 and TNF that stimulate adipose tissue to produce ATX (Figure 3). Another study showed that TME of BC patients has threefold higher levels of ATX than the normal tissue in healthy individuals [133]. Moreover, BC cells favor the binding of ATX to integrin alphaIIbeta3 and alphavbeta3 on the cell membrane, promoting tumor cell migration [133,134] (Figure 3). Therefore, BC accurately represents the correlation between TME and ATX.

### 6.2. Ovarian Cancer

Ovarian cancer corresponds to the second most frequent tumor after BC in females [135]. It has been reported that ATX levels are two-fold higher in ovarian cancer than in normal ovarian tissue [136,137]. Moreover, other studies showed that ATX activity was higher in malignant ascites than serum of patients affected by ovarian cancer [138].

LPA levels were also found in elevated amounts in malignant ovarian ascites [139]. An experimental study reported that the treatment with ATX inhibitors, such as PF8380 or S32826, totally suppressed LPA production by ovarian cancer stem cells (CSCs) with a reduction of CSC features. This suggests that ATX may influence the maintenance of ovarian CSCs employing an LPA-mediated autocrine mechanism [140] (Figure 3).

### 6.3. Hepatocellular Carcinoma

HCC is the most common type of liver cancer [141,142,143]. Some experimental data documented that serum ATX and plasma LPA levels are significantly higher in HCC patients compared with healthy people [144,145]. Studies on mice models of chronic liver diseases demonstrated that various hepatotoxic stimuli stimulate ATX expression by hepatocytes. Consequently, an increase in LPA levels, amplification of profibrotic signals, and activation of hepatic stellate cells (HSCs) were observed. In contrast, the ATX deletion from hepatocytes delayed HCC development [146] (Figure 3).

### 6.4. Pancreatic Cancer

PC is one of the most aggressive cancers with a 5-years survival rate of 5% approximately [147,148,149,150,151,152]. Although the ATX transgenic mouse of the pancreas has not been designed yet, it has been described that the treatment of PC cell lines or rats with pro-oncogenic substances was responsible for increased ATX levels [153]. These data indicate that ATX could be the target of pro-oncogenic substances for PC and, at the same time, suggest that ATX could favor PC genesis [154]. In addition, PC patients have higher levels of LPA in serum and ascites than healthy individuals [155]. Moreover, LPA favored tumor proliferation, cell migration, and invasion in these patients (Figure 3). Therefore, the ATX–LPA signaling pathway might sustain PC development, although the exact mechanisms have not been completely understood yet [153].

### 6.5. Glioblastoma Multiforme

Glioblastoma multiforme (GMB) corresponds to the most frequent and aggressive type of brain tumor. GMB patients have a poor prognosis, with a 5-year survival rate of 5% [156,157]. GMB is characterized by a high recurrence rate due to the infiltrative ability of these tumor cells [158]. Some experimental studies showed that GMB cells have high levels of ATX expression [159]. These data were also reported both in GBM patient samples but also in gliomas of grades I, II, and III, according to WHO. ATX was found in the cytoplasm, both in infiltrative tumor cells and in core ones [158]. In vitro studies on human GMB cells, such as U87 and U251, demonstrated that oxidative stress enhances ATX expression levels, stimulating the invasiveness of GMB cells [160,161]. Moreover, Zhang et al. evidenced that NSun2-mediated ATX mRNA methylation enhanced levels of ATX expression and promoted U87 cell migration [32] (Figure 3).

The employment of ATX-specific inhibitors, such as PF-8380, induced a reduction of cell invasion and migration abilities in vivo and suppressed radiation-induced tumor new vessel genesis. Therefore, ATX inhibition might improve GBM response to radiotherapy [162,163]. Alpha-bromomethylene phosphonate LPA (BrP-LPA) is an ATX inhibitor and an antagonist of four LPARs that proved to reduce the survival and migration of irradiated GMB cells [164,165].

## 7. The Role of ATX–LPA Pathway in Drug Resistance

It has been found that *ENPP2* is one of 90 genes correlated with therapeutic resistance in tumors [166]. Some studies reported that this gene can be most expressed in tumor cells after radio- and chemotherapy [21,167]. 

The ATX–LPA axis was proven to inhibit the extrinsic and intrinsic pathways of apoptosis, prolonging cell survival [168]. In detail, LPA reduces the FAS receptor on the tumor cell surface and, on the other hand, decreases the FAS ligand levels [169,170]. These events lead to a reduction of tumor cell responsiveness to the extrinsic pro-apoptosis stimulation. FAS can also favor intrinsic apoptosis through the activation of intracellular caspases; however, LPA can block FAS-mediated intrinsic apoptosis by the inhibition of caspase-3 and -8 activities [171,172]. Moreover, LPA can also limit intrinsic apoptosis through the increase of Bcl-2 and the suppression of Bax and Bad [171,172].

Several chemotherapeutics, such as tamoxifen, doxorubicin, and paclitaxel, perform by stimulating apoptosis through the formation of ceramides, a class of sphingolipids [173,174,175]. They become substrates of ceramidases to be converted to sphingosine and, consequently, to sphingosine 1-phosphate (S1P) by sphingosine kinase-1 (SK-1). Ceramides are also involved in the initiation of intrinsic apoptosis through the cytochrome C release from mitochondria and the consequent activation of caspases [176]. However, LPA and S1P were proven to contrast the inhibitory activities of ceramides. Moreover, LPA stimulates S1P production in tumor cells prolonging cell survival [177].

The ATX–LPA pathway limits chemotherapy effects on tumor cells. Several studies showed that ATX and LPA favor resistance to platinum-based drugs in colon and ovarian cancer cells [166,178]. In a mouse model of BC, it has been described that combined treatment with doxorubicin plus ONO-8430506, an ATX inhibitor, led to a synergistic anticancer action. Experimental data documented the reduction of plasma ATX activity with this drug. Moreover, it was proven to reduce cell growth of initial BC and subsequent lung metastases by 60% in comparison with control mice. This is probably due to the limitation of Nrf2 stabilization commonly mediated by LPA. Nrf2 is a transcription factor that is induced by LPAR1 stimulation and activation of PI3K [179]. This ATX inhibitor favors the expression of antioxidant genes and multidrug-resistant carriers [180]. In addition, ONO-8430506 has been also studied in thyroid cancer, demonstrating the ability to reduce the tumor volume by 50–60% and the levels of several inflammatory mediators [181].

**Figure 4:**
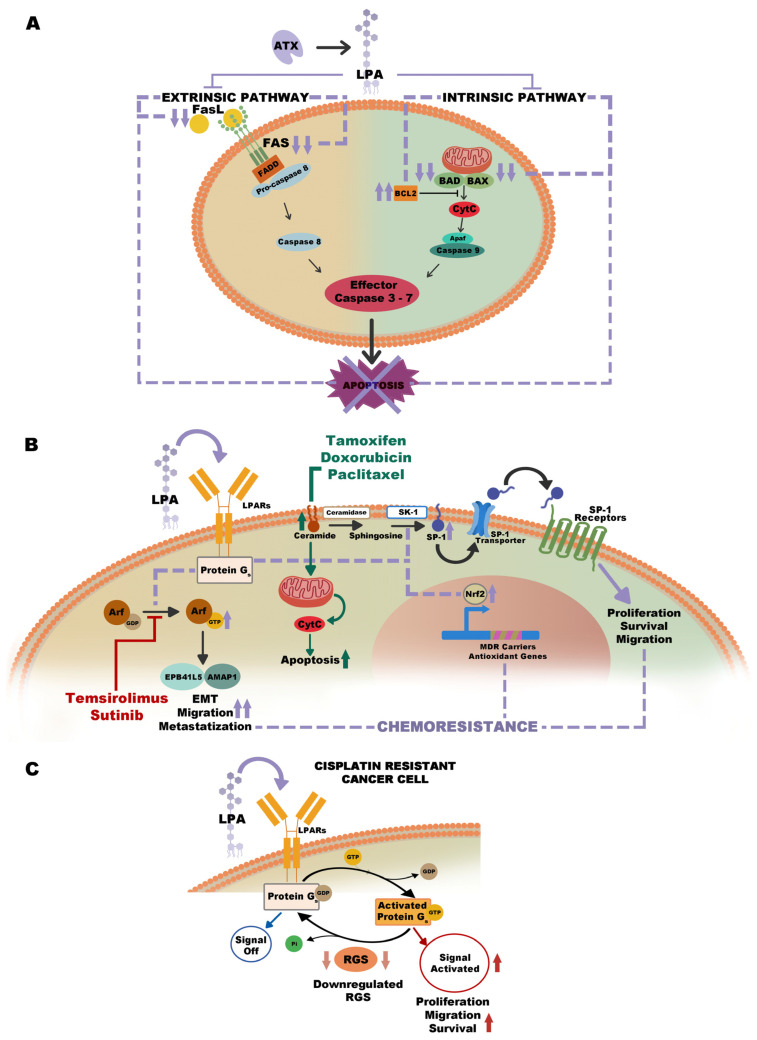
Representation of different drug resistance mechanisms mediated by the ATX–LPA pathway. (**A**) The ATX–LPA axis inhibits extrinsic and intrinsic apoptosis pathways, downregulating FASL/FAS and BAX/BAD and upregulating BCL2. The lilac-colored arrows refer to pathways activated by LPA (**B**) LPA favors resistance to Temsirolimus and Sutinib, activating Arf6 GTPase and its downstream pathway; the ATX–LPA axis stimulates transformation of ceramide in SP-1, countering activities of Tamoxifen, Doxorubicin, and Paclitaxel; LPAR1 stimulates Nrf2, the transcription factor of MDR carriers and antioxidant genes activating chemoresistance. The lilac-colored arrows refer to pathways activated by LPA, the green-colored arrows refer to pathways activated by drugs written in green. (**C**) Downregulation of RGS favors drug resistance in cancer cells by enhancing LPARs activity. Abbreviations: FASL—FAS Ligand; FADD—FAS-associated protein with death domain; BCL2—B-cell lymphoma 2; BAD—BCL2 associated agonist of cell death; BAX—Bcl-2-associated X protein; Apaf—apoptotic protease activating factor; CytC—cytocrome C; LPARs—lysophosphatidic acid receptor; Arf—ADP-ribosylation factor; SK-1—sphingosine kinase-1; SP-1—sphingosine 1-phosphate; Nrf2—nuclear factor erythroid 2-related factor 2; MDR carriers—multi drug resistant carriers; EMT—epithelial-mesenchymal transition; RGS—regulators of G protein signaling.

Another combination with doxorubicin and a novel ATX inhibitor, GLPG1690, was evaluated in a phase 3 clinical study as treatment for idiopathic pulmonary fibrosis [182]. The same combination was tested in a mouse BC model, resulting in increased suppression of cancer growth [183]. ATX and LPA are also responsible for tumor cells’ resistance to other chemotherapeutic drugs. In this regard, it has been described that LPA can activate Arf6 GTPase, favoring the resistance of786-O renal tumor cells to Sunitiniband Temsirolimus. However, it has been also observed that this resistance can be annulled through the knockdown of LPAR2, EPB41L5, or AMAP1, the downstream effectors of Arf6 [184]. An in vitro study on PC cells treated with cisplatin or doxorubicin showed that LPAR3-expressing cells had longer survival than healthy cells. The same cells had higher expression of genes correlated with multidrug resistance. These results suggest the role of LPAR3 in therapeutic resistance in PC [185]. In HCC cells, LPAR6 mediates the resistance to sorafenib by promoting lactic acid fermentation at the expense of oxidative phosphorylation, and treatment with 9-xanthenyl acetate (XAA), a LPAR6 antagonist, can overcome this resistance [186]. Regulators of G-protein signaling (RGSs) proteins may also influence the drug resistance of tumors. In particular, by RGSs limit G protein-coupled receptor (GPCR) activation, such as LPAR, through the increase in the GTPase activity of G-proteins [187]. Experimental data showed that cell lines of cisplatin-resistant ovarian cancer expressed low levels of RGS10 and RGS17. On the other hand, the knockdown of one of these RGS proteins in ovarian tumor cells is responsible for a 2 to 3-fold decrease in the cisplatin potency while the knockdown of both RGS proteins leads to a 6-fold reduction of drug potency. Data on nasopharyngeal tumor cells reported that a high level of RGS17 results in increased sensitivity to 5-fluorouracil [188]. Therefore, the downregulation of RGS proteins might favor the occurrence of drug resistance by enhancing LPAR activity (Table 1, Figure 4).

## 8. ATX Inhibitors and Future Clinical Applications

To date, the treatment of cancer has been based on therapies to counter tumor cell proliferation and survival. However, there is growing evidence that TME components play a key role in favoring cancer development and influencing the therapeutic response. Therefore, these data led to a new research field involving the non-cellular and cellular elements of TME with the aim of removing the sustenance of tumor cells.

Based on the reported data in this review, the ATX–LPA pathway is involved in TME maintenance and plays a very important role in tumorigenesis, neoangiogenesis, cell migration, invasion, metastasis, and chemotherapeutic resistance. Therefore, the inhibition of this pathway might represent a new treatment strategy for tumors by blocking cancer cells and TME elements.

Many ATX inhibitors have been tested in preclinical and clinical trials. GLPG1690 is an ATX competitive inhibitor that blocks LPA production thanks to its capability to occupy the ATX hydrophobic pocket [189]. This drug has been evaluated in phase III clinical studies as a treatment of idiopathic pulmonary fibrosis (IPF) [182,190]. However, more recently, it has been also tested in a mouse model of BC, showing that it is able to significantly reduce tumor cell proliferation and increase radiotherapy effects [183].

PF-8380 has a potent activity of ATX inhibition since it can be retained in the orthosteric site of ATX and mimic the binding of LPC substrate. An in vitro study on human and murine GBM cell lines showed that this drug suppressed tumor cell invasion and increased radio-sensitization through ATX inhibition [162].

RB011 is a DNA aptamer that was proven to inhibit *ENPP1* activity, but it is also able to prevent the access of LPC to the hydrophobic pocket by binding to the ATX active site [191].

HA130 and HA155 are boronic acid-based compounds with the ability to inhibit ATX by binding its catalytic threonine and prevent LPC binding to ATX [191,192].

LPAR antagonists might also have potential efficacy as antitumor drugs, above all for LPAR1, LPAR2, and LPAR3. On the other hand, the inhibition of LPAR4 and LPAR5 could not have a beneficial effect due to their inhibitory activities on tumor cell growth and motility [193]. Several LPAR1 antagonists (e.g., AM966, BMS-986020, SAR100842) have been tested in clinical trials as treatment of non-oncological diseases, such as IPF, with encouraging results [194,195,196]. 

To note, all these drugs aimed to be used as cancer treatment; however, the ATX–LPA axis is involved also in neoangiogenesis, fibrosis development, and therapeutic resistance of tumor and nontumor cells [197]. Therefore, the anti-ATX/LPA strategy could also be employed as a complementary treatment for inhibiting angiogenesis, improving sensibility to chemotherapy, or better controlling adverse events of radiotherapy-induced fibrosis. In this regard, a Phase 1b clinical trial is evaluating the safety and clinical efficacy of the ATX inhibitor IOA-289 alone and in combination with Gemcitabine/Nab-paclitaxel in patients with metastatic pancreatic cancer. Preliminary data indicated that IOA-289 is a novel ATX inhibitor with a unique chemical structure, high potency, and a good safety profile, thus supporting further new therapeutic approaches for cancer treatment, particularly those with a high fibrotic and immunologically cold phenotype (ClinicalTrials.gov ID NCT05586516).

Moreover, it is well known that the LPA/LPAR5 pathway inhibits the cytotoxic activity of CD8+ T cells; therefore, the anti-ATX/LPA strategy might also help to overcome immunotherapy resistance [197]. LPA also plays an important role in cancer-contrasting effects; for example, it is involved in T-cell endothelial transmigration, a crucial process for the intratumor trafficking of these cells.

Hence, the need to design antagonists of the ATX–LPA axis with a target action on those specific LPARs with protumor activity rather than inhibiting all ATX–LPA pathways (Table 2).

## Figures and Tables

**Table 1:** The role of the ATX–LPA pathway in drug resistance in different types of cancer.

Cancer	ATX/LPA Levels	ATX Inhibitors	ATX–LPA Pathway in Drug Resistance	Clinical Application
Breast cancer	- ATX blood levels are higher in BC patients - Threefold higher levels of ATX in tissue microenvironment of BC patients with respect to normal tissue from healthy individuals	- ONO-8430506 - GLPG1690	- Downregulation of Nrf2 transcription factor	GLPG1690 has been evaluated in phase III clinical studies as a treatment of idiopathic pulmonary fibrosis (IPF)
Ovarian cancer	- ATX levels are twofold higher in cancer tissue than in normal tissue - High LPA levels in malignant ovarian ascites	- PF8380 - S32826 - BBT-877	- Enhanced LPARs activity by downregulation of RGS10 and RGS17 - ATX plays a pivotal role in the maintenance of the CSC-like properties of epithelial ovarian tumors (PTX resistance)	------
Hepatocellular carcinoma	- Serum ATX and plasma LPA levels higher in HCC patients	- 9xanthenylacetate	- Resistance of HCC cells to sorafenib promoted by LPAR6 (switch from oxidative phosphorylation to lactic acid fermentation	------
Pancreatic cancer	- LPA levels are higher in serum and ascites in PC patients	- IOA-289	- Role of LPAR3 in expression of genes correlated with multidrug resistance	A Phase 1b clinical trial is evaluating the safety and clinical efficacy of the ATX inhibitor IOA-289 alone and in combination with Gemcitabine/Nab-paclitaxel in patients with metastatic pancreatic cancer
Glioblastoma multiforme	- High levels of ATX in GMB cells	- PF-8380	------	

**Table 2:** Literature overview on the autotaxin–lysophosphatidate axis: promoter of preclinical and clinical studies and key parameters for carcinogenesis and tumor progression.

Research Topic	Setup of Treatment	Cancer Model	Role of ATX/LPA as Biomarker/Therapeutic Target	Role of ATX/LPA in Carcinogenesis	Role of ATX/LPA in Cancer Progression/Metastasis	Role of ATX/LPA in Drug Resistance	Reference
ENPP2 gene expression in breast cancer (BC)	Activation of ATX promoter by hormones	Transgenic mammary glands in mouse models	ATX/LPA overexpression	Induction of precancerous and cancerous lesions	Induction of lymph node metastases	-	[75]
Role of LPAR1/2/3 receptors in: - Pancreatic (PC), ovarian (OC), and liver (HCC) cancer cells, - Hamster pancreatic duct adenocarcinomas (PDAs) - breast cancer (BC) mouse model - BC patients	- LPAR 1/3 knockdown cells - Induction of PDAs by a nitroso compound - Forced over expression of LPAR3 in BC model - LPAR1 inhibition by Bebio-0719 - LPA stimulation of OC cells - Inhibition of LPAR1/3 by Ki16425 in PC mouse models	- PC, OC, and HCC cells - Hamster model - BC mouse model - BC patients	- LPA1 down regulation enhances cell motility - LPA3 down regulation inhibits cell motility - LPAR3 as prognostic marker in BC mouse model - Prognostic value of LPAR3 in BC patients	In PDA models, LPA1 is lower expressed, LPA3 is over-expressed	- LPA1/3 are involved in cell motility/invasion - LPAR3 induces metastasis in BC mouse model - LPAR3 higher expression correlates with higher tumor stage and lymph node metastases in BC patients - LPAR1 expression correlates with BC metastasis - LPAR2/3 stimulated VEGF expression in OC cells and OC biopsies - LPAR1/3 stimulated linphoangiogenesis through VEGFC expression in PC models - LPAR stimulate clycolytic shift in OC models	-	[75,76,79,80,81,83,84,88,89,94,95,99,100]
Role of LPAR6 in HCC patients and xenograft mouse model	RNAi-mediated attenuation of LPAR6	- 128 HCC patient cohort - Xenograft mouse model	- Prognostic role of LPAR6 in HCC patients	Role of LPAR6 in HCC carcinogenesis	-	-	[82]
Role of ATX expression in different cancer models: - PDAs model - Different cancer models (HCC, PC, glioblastoma multiforme (GMB) - ATX overexpression in different human tumor tissues (BC, OC, HCC, PC, glioblastoma multiforme (GMB)) and in malignant ovarian and pancreatic ascites	- ATX-transfected NIH-3T3 cells - ENPP2 knockdown - ATX transfected/RAS-transformed NIH-3T3 cells - IOA-286 ATX-inhibitor - Oxidative stress - Pro-oncogenic substances - PF-8380 ATX-inhibitor - Alpha-bromomethylene phosphonate LPA (BrP-LPA) (ATX inhibitor) - ATX inhibitors (PF8380 or S32826) - Hepatotoxic stimuli	- PDA mouse models - Different tumor cell lines - Mouse models - Human tissues - Malignant ovarian ascites - Human PC serum an ascites - Ovarian cancer stem cells (CSCs) - Mouse models of chronic liver diseases	ATX expression as biomarkers of cancer cell growth - ATX as therapeutic target	- Treatment of PC cell with pro-oncogenic substances increased ATX levels - LPA stimulated profibrotic signals and activation of hepatic stellate cells playing key role in HCC carcinogenesis	- ATX expression in PDA growth and metastasis - ATX expression induced angiogenesis - ATX–LPA axis involved tumor growth and invasiveness - ATX may influence the maintenance of ovarian CSCs by LPA-mediated autocrine mechanism	- ATX-specific inhibitors suppressed radiation-induced tumor new vessel genesis	[32,77,78,129,136,137,138,139,140,144,145,146,153,154,155,158,159,160,161,162,163,164,165]
Role of LPA in fibroblast transformation in OC and HCC models	Tumor-derived LPA	Lung, ovarian, and HCC fibroblast	- Targeted inhibition of LPA-mediated metabolic reprogramming in CAFs	-	- LPA induced cancer associated fibroblast (CAFs) in peri tumor tissues in OC and HCC models	- Reduction of drug delivery to tumor	[85,90,100,117]
Role of ATX/LPA in immunosuppressive microenvironment	- Tumor-derived LPA - LPAR5^−/−^ tumor-specific CD8+ T lymphocytes	- OC patients - Mouse models - Rat models	- Targeted inhibition of LPA-mediated tumor associated macrophages (TAMs) conversion	- ATX/LPA signals are involved in immune escape and carcinogenesis	- LPA induced monocytes conversion into TAMs - LPA-mediated LPAR5 stimulation suppressed CD8+ T cells activation - ATX inhibition decreased allograft infiltration of CD4+ and CD8+ T cells - LPA interaction with LPAR2 on naïve T lymphocytes improved T-cell migration across very small pores		[120,121,123,124,125,126,127]
Role of ATX/LPA axis in drug resistance mechanisms	- Carboplatin - TSA - Radiotherapy - Camptothecin - Cisplatin - Anti-FAS - TRAIL - Etoposide - Cis-Diamminedichloroplatinum - Carboplatin - Doxorubicin+ ATX inhibitor(GLPG1690) - Sunitinib - Temsirolimus - ATX inhibitor (PF-8380) - ATX inhibitor (ONO-8430506)	- OC cells - BC, cervix, lung cell lines - BC patients - Rat intestinal epithelial cells (IEC-6) - CRC cells - Idiopathic pulmonary fibrosis (IPF) patients - BC mouse model - Renal cancer (RC) cells - PC cells - Human and murine GBM cell lines	ATX as therapeutic target: - Doxorubicin+GLPG1690 in BC mouse model and in idiopathic pulmonary fibrosis phase III clinical trial. - ATX inhibitor PF-8380 enhanced radiation effects in human and murine GBM cell lines. - ATX inhibitor ONO-8430506 in BC and thyroid cancers model reduced cell growth and inflammation. - LPAR1 antagonists (e.g., AM966, BMS-986020, SAR100842) tested on non-oncologic clinical trial, such as cutaneous systemic sclerosis and IPF		- ATX over-expression is involved in cancer progression and metastasis	- ATX counteracted apoptosis induced by: carboplatin, TSA, radiotherapy, camptothecin, anti-FAS, TRAIL, etoposide, Cis-Diamminedichloroplatinum, carboplatin - LPA induced genes involved in multi drug resistance - GLPG1690 increased doxorubicin effects in idiopathic pulmonary fibrosis - Knockdown of LPAR2 restored sensitivity to Sunitinib and Temsirolimus in RC model - LPAR3 induced multi drug resistance genes in PC cells - Lower level of RGSs proteins leaded to up regulation of LPAR inducing drug resistance in OC cells	[21,162,166,167,168,169,170,171,172,173,174,175,176,177,178,179,180,181,182,183,184,185,186,187,188,189,190,193,194,195,196]

## Data Availability

Not applicable.
